# Toward Collaborative Intelligence in IoV Systems: Recent Advances and Open Issues

**DOI:** 10.3390/s22186995

**Published:** 2022-09-15

**Authors:** Sedeng Danba, Jingjing Bao, Guorong Han, Siri Guleng, Celimuge Wu

**Affiliations:** 1 School of Computer Science and Information Engineering, Hohhot Minzu College, Hohhot 010051, China; 2 Graduate School of Informatics and Engineering, University of Electro-Communications, Tokyo 182-8585, Japan; 3 B&S Tech Co., Ltd., Arakawa-ku, Tokyo 116-0014, Japan

**Keywords:** internet of vehicles, collaborative intelligence, multi-access vehicular environment, edge computing, machine learning

## Abstract

Internet of Vehicles (IoV) technology has been attracting great interest from both academia and industry due to its huge potential impact on improving driving experiences and enabling better transportation systems. While a large number of interesting IoV applications are expected, it is more challenging to design an efficient IoV system compared with conventional Internet of Things (IoT) applications due to the mobility of vehicles and complex road conditions. We discuss existing studies about enabling collaborative intelligence in IoV systems by focusing on collaborative communications, collaborative computing, and collaborative machine learning approaches. Based on comparison and discussion about the advantages and disadvantages of recent studies, we point out open research issues and future research directions.

## 1. Introduction

**Figure 1:**
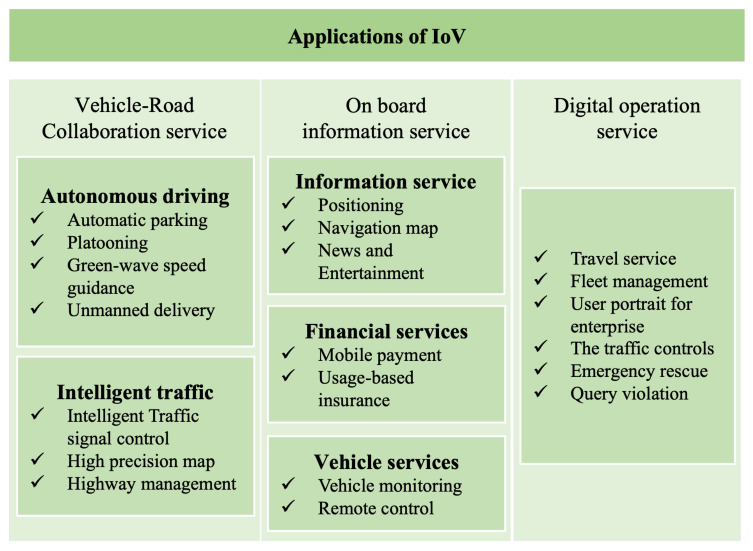
Application of Internet of Vehicles.

As the fourth industrial revolution, industry 4.0 has started an important development stage and academia, industry and management departments are making efforts to realize the intelligent ecology of industry 4.0 [1,2]. As a necessary foundation of industry 4.0 and key use cases such as smart city and smart industry, Internet of Things (IoT) realizes the intelligent perception, recognition and management of objects and processes through the connection between things and things, and people and things. In recent years, Intelligent Transportation System (ITS) and Intelligent Vehicles (IV) are the research hotspots of many researchers and technology companies. As the most important application fields of the IoT, IoV is an essential key technology for the next generation of ITS, and also one of the most important breakthroughs. The realization of IoV can significantly improve the smart city and the sustainable development of energy. For example, the traffic management system in the environment of IoV and vehicles with autonomous driving function can reduce traffic congestion, traffic accidents, and environmental pollution. Vehicles under the networking of an intelligent vehicle system can provide users with more comprehensive and personalized mobile travel service, such as path planning, service recommendation, and intelligent parking to provide a safe, comfortable, intelligent, and efficient driving experience and transportation services, improving the efficiency of transportation. At the same time, they promote an intelligent level of transportation service for society. Applications of IoV are listed in Figure 1. IoV is not only applied to urban traffic, but also has many applications; for instance, internet of underwater vehicles and internet of aerial vehicles. Drones are used in intelligent logistics, oil and gas equipment inspection, environmental and traffic monitoring, disaster recovery and more scenarios. IoV has attracted a large number of researchers and companies due to its great research value and commercial interests.

However, the development of the IoV system still needs to overcome many challenges before it can play a big role because of the above characteristics. These challenges include the following. To turn the "Internet of Everything" into reality in the future, there will be more and more network devices and access environments, resulting in entity heterogeneity, dynamic, data diversity and big data challenges. At the same time, a variety of high-quality new applications, such as autonomous driving and automated delivery and other advanced services, require high network, storage, and computing resources. Therefore, to meet the network requirements of low delay, large bandwidth, secure, and efficient computing requirements of the next generation of IoV, efficiently utilizing the existing limited resources to design the IoV system is a major challenge.

To address these challenges, resource-efficient task processing architecture must to be built while effectively utilizing the limited resources at all levels, such as perception, transmission, and application of IoV. All of these demands drive us to research how these various entities might work together to optimize communication, caching, and computing resources between heterogeneous agents. In the past few decades, AI has developed rapidly and has been successfully applied in many research and application fields. Currently, in the field of IoV, there are many studies showing great interest in AI and collaborative intelligence methods. Collaborative intelligence(CI), also known as cooperative intelligence, is able to aggregate the capabilities and intelligence of multiple devices to achieve a common goal and intent [3].

CI can bring AI to the edge, such as vehicles and roadside units. The lack of sensing, computing, and storage capabilities of edges and endpoints can be solved. Letting a single computing device perform complex tasks alone should be avoided, while the lack of sensing, computing and storage capabilities of edges and endpoints can be resolved. This reduces communication latency and protects data privacy by bringing the AI closer to the user. Some studies have confirmed that the collaboration of cloud and mobile edge can improve end-to-end latency, decrease usage of energy, and increase data-center throughput [4,5].

Many articles have reviewed and surveyed research issues from various perspectives in IoV, including 5G technology evolution [6], edge service optimization [7], attacks and detection mechanisms [8], secure and trust management [9,10,11], and big data extraction and decomposition [12]. There are also articles proposing new generic IoV architectures [13,14], applied Software-Defined Networking (SDN) on VANET [15], or studying the deployment and application of IoV in a smart city [16]. In addition, there are many works on the application of new techniques in IoV; for instance, Deep Reinforcement Learning technologies [17], Blockchain [18], Digital Twins, and Federated Learning [19]. These articles well introduce the basic technology, network architecture, and typical applications of IoV, but rarely involve the problem of collaborative intelligence in IoV. There are still a small number of articles discussing the problem of collaborative intelligence in IoV, including collaboration in smart drones and IoT [20], cooperation and collision avoidance in UAVs [21], and sociality of collaborative CAVs [3], but they are limited to specific fields or research problems, and cannot describe the whole picture of the collaborative problem involved in the heterogeneous network of IoV. In addition, the articles on CI are not limited to IoV [22,23], which is a good reference for the study of CI in IoV scenarios, but more targeted work is desired to discuss CI problems according to the characteristics of fast-moving vehicles and high delay requirements of IoV applications.

The research works discussed in this paper cover a wide range of topics for implementing CI in IoV applications, such as radio resource allocation, routing protocols, data sharing, task offloading, security enhancement, and machine learning methods. We will discuss these topics in three sections: collaborative communication, collaborative computing, and collaborative machine learning approaches. Collaborative communication is the coordinated networking technology, including the routing and access selection in V2R, V2P, and V2V collaborative scenarios, security and privacy during communication, etc. Collaborative computing includes big-data-oriented task offloading and mobile edge computing. Collaborative learning technologies include federated learning, ensemble learning, unsupervised learning, and reinforcement learning.

Due to space and energy constraints, we prioritize research works according to their relevance to the subject under discussion, as well as their quality, location, date of publication, and number of citations. Journal papers published in authoritative journals or magazines and conference papers published in international conference proceedings in the last 3 to 5 years are selected. We also include the most cited papers on collaborative intelligence in the IoV.

The rest of this paper is organized as follows: In Section 2, the concepts and features of IoV and CI are introduced. In Section 3, we describe the collaborative communication technology in the collaborative intelligence of the IoV and then discuss its existing difficulties and existing research in detail. In Section 4, technical issues and existing research in collaborative computing, including task offloading and mobile edge computing methods, are discussed. Section 5 summarizes the development and current situation of collaborative machine learning methods such as joint learning, ensemble learning, unsupervised learning clustering, and reinforcement learning in the collaborative intelligence of the IoV, and analyzes the advantages and disadvantages of each method. In Section 6, we propose the future research directions of CI in the IoV. Section 7 is the conclusion of the paper.

## 2. Internet of Vehicles and Collaborative Intelligence

In this section, the concept, architecture, and characteristics of the Internet of Vehicles are briefly introduced, and then collaborative intelligence and its advantages and challenges in IoV are discussed.

### 2.1. Internet of Vehicles

**Figure 2:**
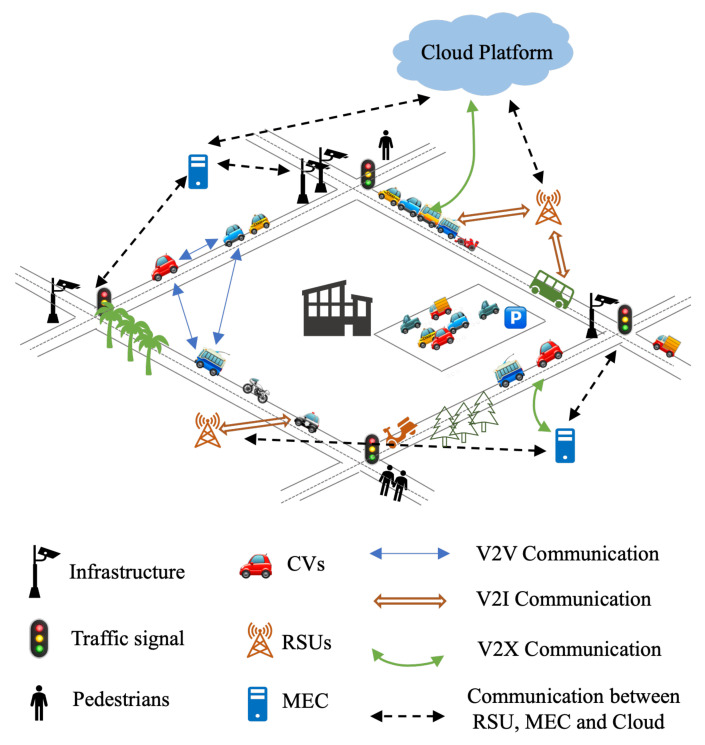
One scenario of Internet of Vehicles.

The IoV is the integration of IoT and mobile vehicle Internet. It is a huge system composed of multiple nodes connected in the network, such as intelligent vehicles driven by humans or autonomous vehicles, infrastructure integrated with intelligent sensing and V2X communication [24], Mobile Edge Computing (MEC) devices [25], and cloud computing service platforms. It relies on multiple disciplines such as networking, communication, Artificial Intelligence, control, social sciences, and cybersecurity to try to provide rich intelligent applications. In general, IoV technology is a dynamic mobile communication system that connects vehicles and connected devices through vehicle to everything (V2X) communication, enabling all necessary devices to share information and interact. It includes vehicle to vehicle (V2V), vehicle to road (V2R), vehicle to infrastructure (V2I), vehicle to pedestrian and cyclist (V2P), and vehicle to cloud/edge (V2N) communication [24]. The scenario is shown in Figure 2. An IoV system can supervise and guide traffic efficiently in real time and provide more intelligent mobile services through data collection, sharing transmission, storage and computing and analysis capabilities.

**Figure 3:**
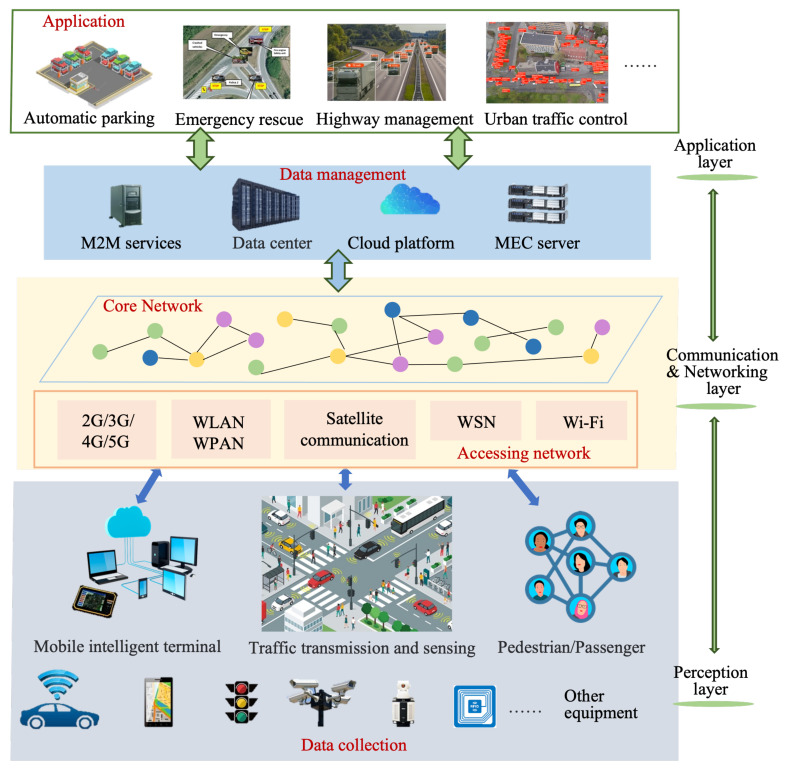
Three-layer Structure of Internet of Vehicles.

The traditional IoV architecture is usually divided into three layers, including the perception layer, the network layer, and the application layer. According to the specific application scenarios or refinement functions, many studies have divided the IoV into four layers [26], five layers [13], seven layers, and other different levels of architecture [27]. Here, we still consider IoV as a three-layer structure, as shown in Figure 3.

The perception layer is the foundation of IoV and contains all sensors inside and outside the vehicle, which collect environmental data and detect specific events of interest; for instance, vehicle condition, road conditions, traffic lights, and environmental conditions. The goal is to obtain the knowledge of the whole environment by collecting information from multiple angles [28]. After screening, integration, and analysis, these large amounts of collected information can afford data support for the realization of various businesses in the IoV.

The second layer is the networking layer, or communication layer, which supports different V2X wireless communication modes. It allows seamless connectivity with existing and emerging networks such as GSM, Wi-Fi, LTE, Bluetooth, 802.15.4, 5G/6G, etc. This is so that the large amount of data and signals generated at all times can be transmitted everywhere without delay, while minimizing the resource consumption. At present, the main communication standard of IoV is Dedicated Short-Range Communications (DSRC) based on the IEEE 802.11 standard, which is led by the United States and cellular-vehicle to Everything (C-V2X) which is the global unified standard communication technology [29].

The application layer includes statistics, storage management, computing, and processing infrastructure, which provides the analysis, processing, and decision-making of different situations based on big data for ordinary users, management departments, and enterprises. The goal is to be able to fuse information from different systems and technologies to make a unified decision. It involves the allocation of computing tasks and resource management [30].

Internet of vehicles is widely used. At present, research on IoV is mainly focused on low-delay information transmission, traffic safety and efficiency, high reliability, and other aspects, and oriented to big data [27]. In addition, security and privacy protection is an important part of every layer of IoV. Omnidirectional sensing and data sharing require user privacy protection and system security to be in place, so there are many related studies [31,32].

Some features of IoV are as follows:

- **Information gathering:** Data are collected through the use of various sensors (pollution detection sensors, cameras, road sensors, etc.) that provide drivers with enough information to react to environmental changes in an adequate and effective manner.
- **High mobility:** Vehicular networks (VNs) have highly mobile nodes, related to the speed of the car, and nodes can be added and disappear in a short period of time, resulting in repeated topology changes.
- **Type of information transmitted:** Messages are transmitted according to the level of participation in the event triggered. Messages can be sent from an initiator to a destination (unicast), or from an initiator to a specific cluster using multi-hop communication (multicast). Vehicles can also send messages to all other vehicles via broadcast.
- **Processing big data:** A large number of VNs generate large amounts of data, and vehicular networks use cloud computing to process and store big data.
- **Internet facilities:** The IoV has the unique feature of accessing the Internet. The connected vehicles can benefit from this huge network.

### 2.2. Collaborative Intelligent in IoV

Collaborative intelligence is capable of merging the data, power, and intelligence of multiple pieces of connected equipment in order to accomplish a shared objective and purpose. In the IoV system, this usually manifests as real-time communication and interaction, organic combination of data, sharing of computing and storage resources, and synchronization and scheduling of network access IoV entities, including intelligent vehicles, roadside units, users and mobile devices, edge computing servers, cloud computing centers, data centers, etc. In this framework, AI computation and inference will be deployed simultaneously on edges and the cloud. One possible structure is that the front end of the AI model is deployed on edge devices and performs the initial processing and feature computation. Then, intermediate features are sent to the cloud, and the AI back end completes the step of inference [22].

**Figure 4:**
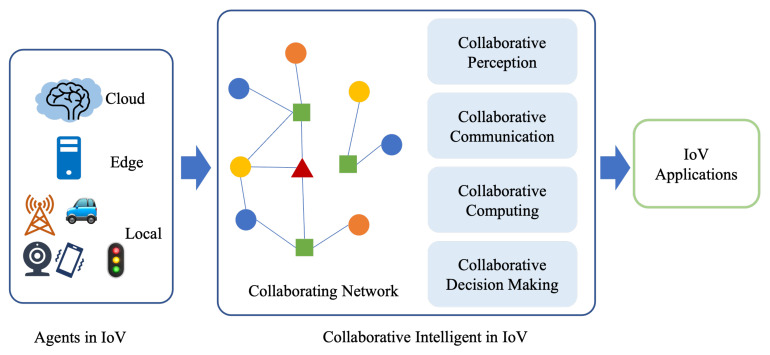
Collaborative Intelligence in the Internet of Vehicles.

As shown in Figure 4, ideas and methods of CI can be integrated into all aspects of IoV. Through cooperation between communication-related smart devices, agents are connected to form a heterogeneous collaborative network, so as to carry out collaborative sensing, collaborative communication, and collaborative computing on this network, in order to make collaborative decisions.

Since AI-based CI methods can produce superior decisions with lower communication overhead by aggregating knowledge and achieving efficient coordination among multiple agents, CI is an inevitable development trend of IoV systems. Specifically, it has the following advantages:

- Collaborative communications over various wireless spectrum types among many transmitters to increase the efficiency of the spectrum utilization.
- Collaborative computing with an end-edge-cloud task processing framework that is resource efficient, meeting a variety of demands on the massive amount of real-time data processing, including exceptionally high throughput and ultra-low latency.
- Collaborative caching among numerous network entities to decrease service latency.

Despite the many benefits of CI, such as communication and computation benefits, it also introduces new challenges in practical applications, so new scientific and technological theories need to be established to attain the optimal design. These challenges include:

- Enable coordination between smart devices.
- Privacy protection and data security issues arising from data and information sharing.
- Efficient collaborative learning with low overhead in scenarios with limited bandwidth and strict latency requirements.

## 3. Networking Technologies toward Collaborative Intelligence

Internet of Vehicles is a large system network composed of an intra-vehicle network, an inter-vehicle network, and a vehicle–cloud network for radio communication and information interaction. Through the integration of the three networks, V2X communication can be more seamless, more efficient, and less communication dead zone. However, with the development of the IoT and vehicle-related technologies and the increasing demand for new service applications, more and more devices are being connected or are ready to be connected to the network. This requires the transmission and exchange of a large amount of data to achieve the purpose of sensing, transmission, and application. ITS has been revolutionary to change, and every device has the potential to become an intelligent node in IoV to participate in collaboration. This results in the transmission of large-scale signals and data, which must rely on stable, trusted, low-latency communication technology. At the same time, the diversity of access devices and communication approaches, the high dynamic and mobility of vehicles, and frequent topological changes in IoV pose challenges to V2X communication.

**Figure 5:**
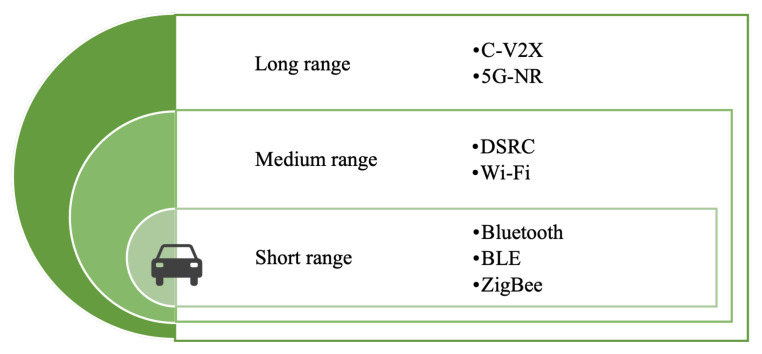
Taxonomy of IoV communication technologies with communication range.

Considering CI, networking technologies in IoV are classified from multiple perspectives below:

- **From the perspective of communicating object**
  It can be divided into V2V, V2I, V2P, and V2N, such as the urban traffic scene in Figure 2. Through V2V communication, the information of the surrounding vehicles can be obtained in the process of vehicle driving, and the vehicles can also constitute an interactive platform, which is often used to transfer control information or safety information between vehicles. Generally, the time delay requirements are high. V2I allows vehicles to communicate with roadside infrastructure (such as traffic cameras, roadside units, bus stations, traffic lights, and parking lots), and through the roadside infrastructure, vehicles can also obtain information about nearby vehicles and send various real-time information. Usually, the amount of data transmission is large, which is mainly used in real-time information service and vehicle monitoring and management. V2P involves communication between vehicles and mobile devices used by vulnerable traffic groups (such as pedestrians and cyclists). V2P communication can be implemented via Bluetooth or Near Field Communication (NFC) technology, and it is often used to avoid traffic accidents and information services. V2N is the connection and information exchange between vehicle or driver and cloud platform or internet through an access network/core network. After storing and processing the acquired data, various applications and services are provided for vehicle users and management departments [33].
- **From the perspective of communicating range**
  Communication in IoV can be classified as short-range, medium-range, and long-range communication [34], as shown in Figure 5. Short-range communication technology can be used for in-vehicle device connection or V2P scenarios, including Bluetooth, UWB, ZigBee, etc. At present, Wireless Local Area Network (WLAN) and DSRC are able to provide wireless communication within approximately 300 meters for IoV, which can be used in V2V and V2I scenarios and classified as medium-range communication. Cellular Communication technology enables vehicles to communicate with objects thousands of meters away. Nowadays, there is no one communication method that can meet all the communication needs of IoV. Therefore, how to select and formulate a scheme to ensure the CI between vehicle and vehicle, and vehicle and environment, is a key concern when the above multiple access methods coexist.
- **From the perspective of data dissemination and protocol**
  It is also possible to classify the network technologies of IoV into three categories: unicast communication, geocast, and broadcast communication, which respectively represent from one vehicle to another, from a vehicle to a group of vehicles, and from one vehicle to all other vehicles in the specified range. Important data can be transmitted in a dedicated resource pool using unicast transmission to avoid data transmission, such as platooning and advanced driving. For sensor data sharing or geographical location demanding scenarios, the geocast or broadcast transmission were carried out through the shared resource pool. Broadcast communications are used when collision warnings and traffic jam messages are disseminated [35]. From the perspective of routing, there are single-hop and multi-hop communication methods [36].

Collaborative communication and networking technology is the key to achieving CI tasks between heterogeneous agents in IoV. This section mainly focuses on three different broad topics, namely Radio Access Technology (RAT) selection, routing protocol, and authentication and secure communications in IoV.

### 3.1. Radio Access Technology Selection in IoV

As mentioned above, there are many types of communication methods that can be used for IoV, and they cannot be replaced with each other. At the same time, the bandwidth demand of users is also increasing, so wireless networks coexist to provide the best service. Vehicles can use different RATS to communicate with other vehicles, infrastructure, networks, etc. Therefore, it is necessary to select the best communication mode and reduce the waste of resources in the multiple access network coexisting environment to ensure cooperation [37].

The RAT selection method can have two schemes [38]. The centralized approach considers the needs of all users in the network and maximizes the throughput or minimizes the delay to centrally optimize the network task. The distributed approach is one in which mobile users improve their own performance as much as possible and regardless of global collaboration, generally using heuristic methods. RAT selection problem is usually treated as a multiple-attribute decision-making problem, or one which uses AI-based algorithms such as neural networks and Q-learning methods. Table 1 summarizes the recent studies on the RAT selection in IoV system.

**Table 1:** Recentstudies on RAT selection in IoV.

Publication	Research Summary	Communication Technology	Scenarios
[39]	A network selection approach that The dynamic Q-learning algorithm was used to verify the necessity of handover, and the fuzzy CNN was used to select the network.	DSRC, 4G-LTE and 5G mmWave	V2V
[40]	The communication performance of DSRC and LTE in three typical IoV applications is tested in a real road environment.	DSRC, 4G-LTE	V2V, V2I
[41]	An Integrated Approach of 4G LTE and DSRC for IoV by Using a Novel Cluster-Based Efficient Radio Interface Selection Algorithm.	DSRC, 4G-LTE	V2V
[42]	A heterogeneous network architecture incorporating multiple wireless interfaces and a Best Interface Selection algorithm are proposed.	WAVE, Wi-Fi, 4G-LTE	V2I
[43]	A conceptual multi-RAT OBU architecture for personal mobility vehicles such as shared bikes, segways or electric scooters and its RAT selection approach are discussed.	LPWAN, Wi-Fi, Cellular	V2I, V2N
[44]	A vertical handover architecture inside the vehicle is implemented based on the logical interface in TCP/IP mode.	IEEE 802.11P, cellular, Wi-Fi	V2X
[45]	A Dynamic Radio Access Selection and Slice Allocation algorithm for 5G and above heterogeneous networks is proposed.	5G and above: small-cells, macro-cells, Wi-Fi	IoT

Hussain et al. [39] studies the network selection and routing when different communication modes exist at the same time, including DSRC, 4G LTE, and 5G mmWave. In terms of network selection, the Shannon entropy rule is used to dynamically set the signal strength, and dynamic Q learning is applied to decide whether the communication mode has to be switched. At the same time, a total of 32 fuzzy rules are formulated after considering factors such as distance, line of sight, vehicle density, signal strength, and data type, and then a fuzzy convolutional neural network (CNN) is adopted to execute RAT. In the aspect of routing between vehicles, the objective function based on channel and vehicle state is designed, and the jellyfish optimization algorithm is improved to calculate the routing.

There are also many studies comparing DSRC, Wi-Fi, and cellular factors. In Ref. [40], the communication performance of DSRC and LTE in several application scenarios of IoV is discussed, and corresponding software and hardware are developed for verification. The results show that 4G LTE is more suitable for non-secure applications with low communication speed requirements such as internet access, traffic data transmission, and file download, while DSRC performs better in secure applications such as traffic signs and collision avoidance. Finally, it is suggested to combine the two methods to handle communication problems in IoV, so as to better support high-quality remote information transmission services and security services.

On the premise of studying the advantages and limitations of DSRC and cellular network technology, Hussain et al. [41] proposes an integration scheme combining these two potential networking technologies to complete more efficient vehicle communication. In this paper, an algorithm combining DSRC and 4G-LTE with a new data forwarding protocol based on optimal clustering is proposed. This algorithm is used to intelligently monitor and evaluate the level of packet loss rate to reduce the handover delay.

In Ref. [42], Sherazi et al. proposed a heterogeneous structure that fully integrated several wireless interfaces, along with WAVE, LTE, and Wi-Fi, by utilizing a radio approach for network access. Furthermore, this architecture was proposed to meet requirements such as pervasive connectivity in order to make VANETs adaptable and expandable for IoV to support a wide range of emergency services. This architecture also used the Best Interface Selection algorithm to ensure reliable communication by using the best wireless interfaces to provide suitable connectivity required for successfully forwarding data in vehicular networks in order to avoid single point failure. The results demonstrated the method’s suitability in IoV. Vehicle classification based on application needs is advised in future research in order to avoid access control issues with increasing vehicles and demand.

Sanchez-Iborra et al. [43] considers the incorporation of new environmentally friendly mobile devices, such as motorcycles or bicycles, into collaborative intelligent transportation systems, and a variety of communication techniques—for instance, vehicular Wi-Fi, low-power WAN, and cellular networks—are already available. These communication methods, however, are not fully covered and do not meet the demands of energy consumption and quality of service (QoS). As a result, they propose a decision support frame by applying supervised ML classification to select the best transmission interface in the multi-radio access technology (RAT) device to send a specific message. Various ML algorithms are investigated while considering the computational and energy usage of IoV terminal devices and traffic types. Their method is tested using a decision tree-based microcontroller unit decision support system, and it demonstrates that it can save energy while performing communication tasks and meeting the QoS requirements of some urgent messages.

Vertical handover refers to switching among two distinct networking techniques, while horizontal handover refers to handover among distinct cells of a single network technology. Tuyisenge et al. [44] studies a mobile internal vertical handover mechanism in a Heterogeneous Vehicle network (HVN) with IEEE 802.11P, cellular network, and Wi-Fi. They suggest a vertical handover management strategy that cuts down on setup time to speed up binding updates. By simultaneously connecting to several available networks utilizing the PMIP-HD architecture, this method enhances handover outcomes by predicting the subsequent handover. It uses a proposed logical interface to profit from the connectivity session. The performance of the proposed approach is verified through stimulations of packet delivery rate, packet error rate (PER), handover connection duration and latency, jitter, and throughput.

In tedious scenarios such as the future IoV, the network slicing paradigm has been studied as technology used to solve the dynamic, isolation, and programmability of mobile networks [46]. This is the layering of an infrastructure into many logical network slices (NSs) so that intelligent systems can flexibly allocate system resources to these network slices based on requirements and network conditions. González et al. [45] proposed Dynamic Radio Access Selection and a Slice Assignment algorithm suitable for a heterogeneous network at 5G or above. Multi-attribute decision making and analytical hierarchy process are used in the selection process, which takes into account multiple entities in a network. Their approach handles overload using collaborative game theory, allowing slices to be reallocated on demand and accepting more users with sufficient service awareness. The distinction between premium and ordinary customers ensures that high-priority users receive excellent service quality. The algorithm combines SDN and network function virtualization methods to provide a comprehensive solution for ratio access selection and slice allocation. Since the above methods are proposed in IoT scenarios and slow mobile users are the experimental objects, whether they can also achieve good results in high-speed mobile IoV scenarios needs further experimentation and discussion.

### 3.2. Routing Protocol in IoV

In recent years, a lot of research of IoV has been carried out on the communication design in the routing protocol. The papers discussed here are listed in Table 2. One type of routing protocol is location based. It designs communication schemes based on location-related information, such as geographical location, topology, and maps [47,48]. In Ref. [48], Ghaffari et al. propose a VANET location-based hybrid opportunistic routing protocol that effectively addresses the location of nodes, as well as the link quality and density of nodes. This process utilizes a greedy forwarding scheme in which the sending vehicle selects the neighborhood with the greatest geographic progress. A mechanism for identifying and remove expired nodes in the routing process has been added, which uses information such as moving direction, velocity, and distance between nodes to measure the lifespan of links. The best candidate node is chosen based on the opportunity and location strategy, and the appropriate data transmission priority is set. Performance is improved in packet delivery ratio, throughput, and end-to-end delay. In Ref. [49], a multi-hop communication protocol based on location information and fuzzy logic is proposed. In Ref. [50], a mobility-prediction-based routing protocol is discussed to lengthen the network lifespan and shorten vehicles’ end-to-end delays. In Ref. [51], the intention of the driver in the vehicle positioning system is used for neighborhood detection, packet transmission, and path recovery in VANET, and a routing protocol based on movement prediction is studied.

**Table 2:** Recent studies on Routing Protocol in IoV.

Publication	Research Summary	Communication Technology	Scenarios
[47]	A survey about geographic routing protocols of three type of VANETs: Delay Tolerant, Non-Delay Tolerant and hybrid type.	Position based	V2V
[48]	An opportunistic and position-based routing protocol with candidate relay node set selection strategy, the priority scheduling scheme and removing the expired links mechanism.	Opportunistic and Position based	V2V
[49]	A multi-hop greedy position-based routing algorithm with fuzzy logic techniques is proposed.	Position-based routing with greedy fuzzy logic	V2V
[50]	A mobility-aware dynamic-clustering-based routing which forms clusters based on Euclidean distance, uses a Mayfly optimization algorithm to select cluster heads and forwards data to RSU is proposed.	Position-based clustering method	V2I
[51]	A routing protocol based on movement prediction is studied using the intention of the driver.	Position based	V2V
[52]	An improved position-based routing protocol with a Kalman filter and an extended Kalman filter is studied.	Position based with Kalman filter	V2V
[53]	A routing protocol considering network connectivity to dynamically clustered vehicles and select gateway nodes is studied.	Connectivity prediction-Based clustering	V2V
[54]	Proposed a vehicle-density-prediction-based routing protocol in which optimal relay nodes are selected on the road grid according to the real-time traffic information.	Grid and vehicle-density- prediction based, neural network	V2V
[55]	A method of actively selecting routing and communication interface with Q-learning is proposed in the case of multi-access vehicular edge computing environment.	Reinforcement-learning based	V2V, V2I

However, the location-based protocol still has some limitations. As the conditions of the traffic environment are changing and becoming more and more complex, the communication links are frequently disconnected because of the mobility of vehicles. Therefore, the communication scheme based on the location information alone cannot fully reflect the communication status between vehicles. Therefore, some studies use a Kalman filter and an extended Kalman filter to improve location-based routing protocols [52], or consider network connectivity to dynamically cluster vehicles, and select gateway nodes in the cluster for communication routing [53]. The reliability of these methods is poor when the distribution of vehicles is uneven, and the effect is not ideal because the scope to be covered is too large and the global structure of the network is not fully structured.

Liu et al. [54] studies a routing protocol named VDPGrid using vehicle density prediction. The vehicle density prediction model uses CNN and long short-term memory (LSTM) to mine spatial and temporal related characteristics in an urban trajectory dataset. The results of the above prediction model are then combined with link quality and path length to assign a weight to each path. In order to reduce communication overhead and computational complexity, after dividing the map into multiple grids, the optimal routing paths are stored in packets, so that data packets can be forwarded between different grids according to road weights. The established vehicle trajectory prediction model can select strategies based on different relay nodes of vehicle cooperation and allocate optimal paths to transmit data packets. This method has less overhead and avoids the grid with poor communication conditions when considering grid selection, so it has better performance in terms of delivery rate. In the process of path selection, the collaborative decision mechanism is adopted, and the overall performance of the method is good.

In Ref. [55], route selection in the edge computing of multi-access vehicles is discussed. The main idea is to learn route selection using the Q-learning method in reinforcement learning, and to actively select routes and communication interfaces to reduce the cost of finding routes after receiving communication requests. A reward rule is designed to learn the route selection method from the feedback of cloud or communication partners and the feedback received by the next hop during the learning process, which is combined with the collaboration of end-edge-cloud and different QoS requirements of delay-sensitive and traffic-intensive applications. Vehicular edge selecting scheme based on fuzzy logic methods is also designed. It can meet the throughput and latency requirements of applications with varying QoS requirements.

### 3.3. Authentication and Secure Communications in IoV

More and more connected devices are joining the IoV. The comprehensive application of collaborative intelligence in the IoV requires a variety of devices to take part in the data collection, transmission, and calculation processes. This leaves the communication process between vehicles, users, Road Side Units (RSUs), and edge and cloud servers all exposed to network security attacks. This is a very serious problem because it involves a large range of traffic safety issues, and may endanger lives. For example, critical information such as traffic jams, traffic light information, accident reports, and the driving status of nearby vehicles may be intercepted and modified by malicious vehicles or operated RSUs, resulting in missing or incorrect information that can cause damage or accidents. Therefore, protecting the critical data communication in the network is a key link in IoV. In addition to data communication security, another important issue is user privacy protection. The recent studies in authentication and secure communications are listed in Table 3. There are many forms of attacks from all sides in the IoV. In article [56], 14 possible attacks on vehicular ad hoc networks are listed. These include Greedy Drivers, Impersonation Attack, Wormhole Attack, Pranksters, Blackhole Attack, and Denial of Service. Therefore, high requirements are put forward for the IoV system.

A collaborative intelligent vehicle internet environment, from the perspective of privacy and security, must be able to [57]:

- Achieve effective and fast authentication of the validity and integrity of messages.
- Avoid exposing user privacy to anyone other than the licensor.
- Trace the appearance of malicious nodes or false information in the network.
- Ensure that the information transmitted by malicious nodes is irrelevant to the control of vehicles.
- Withstand cryptanalysis attacks by quantum computers.
- Prevent middleman modification, counterfeiting, authentication table theft, replay attacks, etc.

**Table 3:** Recent studies on Authentication and Secure Communications in IoV.

Publication	Research Summary
[56]	The security and privacy issues in VANETs are reviewed.
[57]	A quantum-defended blockchain-assisted data authentication protocol is proposed.
[58]	An effective vehicle-centric CRL distribution mechanism is proposed for secure and privacy-preserving IoV.
[59]	A blockchain-based data exchange system that is safe and verifiable is investigated.
[60]	A bivariate polynomial lightweight mutual authentication and key agreement protocol with blockchain is proposed.
[61]	An authenticated key agreement protocol without bilinear pairing is proposed to meet the security requirements of a fog-based vehicular network.
[62]	A secure authentication key management protocol for the deployment of IoV based on fog computing is studied to realize the safe communication in vehicle network, RSUs, fog, and cloud servers.
[63]	A framework that uses decentralized off-chain dataset and blockchain networks is proposed to increase the security.
[64]	A blockchain-based payment strategy for intelligent vehicle refueling to protect sensitive information when data sharing.
[65]	A trusted routing scheme based on blockchain and fuzzy logic is proposed to improve discrimination of malicious user in vehicular network.

Secure communication protocol has always been an active research field. With the development of IoT technology, security authentication protocols in IoV are also developing. In view of the above safety requirements, researchers have carried out many related studies recently. To solve the problems of vehicle identity privacy, distribution of certificate revocation Lists, and computing and communication constraints of vehicular devices intermittently connected to infrastructure, Khodaei et al. [58] designed certificate-based batch authentication technology. However, the fast data communication between vehicles in such schemes and RSUs requires cumbersome certificates, and has a high key storage burden and a long operation time.

In view of the user’s computing power and the sensitive information contained in the access policy, Fan et al. [59] designed an attribute-based ciphertext policy encryption technology for vehicle authentication, and supported information cancellation of vehicles that no longer shared data.

In Ref. [60], a bidirectional authentication scheme based on decentralized key management is designed to automatically enroll, refresh, and withdraw the customer’s public key. But these two methods are vulnerable to quantum attack.

The IoV based on fog clouds is another variant of mobile cloud computing. There are many studies that try to combine fog computing with VANETs to satisfy the requirements for mobility and low latency in practical VANETs. Ma et al. [61] proposed a novel protocol with authenticated key agreement and without bilinear pairing, which realized mutual authentication, generated session keys with security conventions for secret communication, and supported privacy protection. Wazid et al. [62] proposes one lightweight authenticating and key-managing scheme for IoV based on fog computing, which implements authentication key management between vehicle and fog server, RSU and fog server, and cloud server and fog server.

Recently, blockchain technology has been used in many fields as a p2p decentralized computing architecture. In IoV, it is a distributed database retained by multiple entities in the network that can serve as a consensus, privacy, and security protocol to protect V2X communication.

For traffic control of flying vehicles, Allouch et al. [63] presented the blockchain-based lightweight security system UTM-Chain. The visited flight plan or collection of waypoints is saved by the vehicle in a transaction, which is then verified and stored in a block to securely manage vehicle flight. The blockchain is updated with these blocks using cloud servers. A decentralized database is adopted to maintain encrypted vehicle driving records to secure the privacy of information, as well as to lighten the load on the storage capacity of the cars. The system is resilient to different threats and is easily adaptable to the IoV environment, according to the performance evaluation.

To provide clarity, privacy, and trust while erasing any human contact, Jamil et al. [64] discuss a new trading tactic for intelligent vehicle refueling with blockchain. The solution is implemented using a hyperledger structure and is designed so that data sharing between different participants and intelligent vehicle is not allowed.

In Ref. [65], Inedjaren et al. propose a secure and trusted routing scheme for VANET based on blockchain and fuzzy logic. Using blockchain, malicious vehicles’ information can be shared and removed. In order to avoid the burden of a proof-of-work blockchain consensus algorithm, this paper proposes a trust-proof consensus algorithm suitable for a VANET distributed, dynamic, and constrained resource environment. This scheme calculates the trust degree of nodes according to their routing performance. This method uses the Optimized Link State Routing routing protocol and is therefore only used to eliminate the case of malicious vehicles. At the same time, the system overhead is higher than that of other methods.

Gupta et al. [57] designs a new data authentication protocol without a certificate, which is a conditional privacy protection authentication method based on lattice, and realizes the security characteristics of open wireless communication in the IoV. Quantum attacks can be reduced or avoided by using lattice cryptography. On this basis, a reliable blockchain mechanism is proposed, which provides a basis for batch data verification of vehicle reliability and can maintain vehicle anonymity at the same time. In the ideal environment can achieve better communication storage and energy overhead.

The energy consumption and burden of future IoV will be increasingly high, so energy efficiency will be another key issue, and there are quite a few research studies that highlight this [66]. Wang et al. [67] discussed future communication and energy management issues in IoV and proposed an energy-harvesting framework and strategy based on V2I communications to maximize the utility of battery-enabled RSUs and EVs in a V2I communications environment. Cesarano et al. [68] studied the dynamic allocation of RSUs considering the energy consumption and transmission cost of dynamic communication connections between vehicles and RSUs. Sodhro et al. [69] focused on the optimization of QoE and energy consumption in multimedia communication in IoV, and proposed a cache allocation algorithm based on artificial intelligence. As the focus of the sustainable development of green IoV, energy management should be integrated into every link of the IoV system.

## 4. Collaborative Computing in IoV

Emerging applications in the IoV are committed to providing users with efficient, comfortable and secure services and experiences, which requires not only efficient and stable networking and communication schemes, but also more and more computing and storage resources. This is especially true in the IoV with collaborative intelligence applications. While on-board computing and storage capabilities of connected vehicles have been growing rapidly, they are still insufficient for computationally intensive and delay-sensitive tasks. For this purpose, computing tasks are transferred to other nodes with abundant computing resources, such as utilizing cloud computing. However, the centralized cloud computing method shows unstable connection and long propagation delay in the face of the high-speed dynamics of vehicles in the IoV. Even if 5G communication is used, there will be a non-negligible delay. Mobile edge computing, or multiple access edge computing (MEC), has been introduced into IoV to address the problems associated with delay-sensitive applications [70,71].

In the big data environment of IoV, the current solution trend is to consider vehicle–edge–cloud computing collaboration to deal with a large number of complex computing tasks and quickly obtain results. Through the collaboration between the edge and the cloud, the vehicle can transfer the low-latency or low-computation-intensive tasks that were originally processed by the cloud to the edge near the vehicle to compute. In this way, the computing and storage resources of edge devices can be reasonably utilized while reducing the communication overhead. There are many research directions in the collaboration of vehicle, edge, and cloud, such as resource scheduling problems [72], the computing-task offloading problem [73,74], data security [75], the privacy protection problem [76], etc. In addition, studies have discussed the improvement in communication modes and the optimization of perception after the introduction of edge devices into IoV. In article [77], a vehicle clustering algorithm is proposed for routing selection and communication while edge computing devices are deployed in RSU. Dai et al. [78] discusses the problem of object detection under the edge computing framework.

In IoV multimedia applications, Xu et al. [79] discuss how to manage content caching on edge devices according to service requirements to ensure resource efficiency and quality of service, and propose an edge content caching scheme. Based on edge computing, combined with blockchain, Bayesian, and other technologies, Xiao et al. [80] study the problem of fake news detection in the IoV. Xu et al. [81] study estimation of the amount of multimedia services offloaded on edge servers in the future to accurately schedule and reserve the resources of edge devices.

In this section, we will focus on computing task offloading and storage resource management. These two parts are the most important and must be solved in the collaboration of vehicle, edge and cloud. Therefore, we can classify these existing works on collaborative learning technologies into the following two aspects for discussion, as shown in Table 4.

**Table 4:** Recent studies on Collaborative Computing in IoV.

Purpose	Publication	Research Summary
Task offloading	[82]	The cloud–MEC collaborative computing offloading problem is established by co-optimizing computing offloading decision and computing resource allocation.
	[83]	Intelligence-Sharing MEC framework with aggregation and representation for context features, relationship mining and reasoning, and knowledge transfer among MEC servers is discussed.
	[84]	A deep cooperative hierarchical end–edge framework using data communication, computation offloading, and content caching is proposed.
	[85]	An edge caching and computing management problem that jointly optimizes service caching, request scheduling, and resource allocation policies is proposed.
	[86]	To balance delay and energy usage, a distributed iterative approach to handle multivariable and time-varying channel conditions for computational offloading methods is proposed.
Data storage and cache management	[87]	A cache service registry on mobile entities and a new metric to evaluate the effectiveness of service discovery are proposed.
	[88]	A collaborative edge caching scheme is proposed, which shares communication, computation, and caching, and co-optimizes content placement and delivery through flexible trilateral cooperation among macro cells.
	[79]	An edge content caching method for service demand prediction in the IoV in smart city scenarios is proposed.

### 4.1. Big-Data-Oriented Task Offloading

In MEC architecture, the computing tasks of nodes such as vehicles and RSUs are offloaded to nearby devices with idle resources as much as possible. Zhao et al. [82] proposed a collaborative method based on MEC and cloud computing to offload services to cars in vehicle network, which means using cloud resources to provide more computing power to vehicles. By co-optimizing computing offloading decisions and computing resource allocation, the cloud-MEC collaborative computing offloading problem is established. In the Collaborative Computing Offloading and Resource Allocation Optimization scheme, a distributed computing offloading and resource allocation algorithm is designed to achieve the optimal solution, which allows users to request CC resources through their MEC links instead of letting edge servers make decisions. While it can respond quickly when resources are scarce, it is not well suited to tasks with high computational or storage requirements.

The framework proposed by Guo et al. [83] can share the intelligence of different MEC servers to improve their performance. By using MEC and knowledge transfer, the processing speed and accuracy of object detection in different vehicle network scenarios can be significantly improved.

In Ref. [84], it was stated that collaboration among network users can allow them to pool their sensor data and compute results. This eliminates the need for them to manually perform various tasks and increases the efficiency of their networks. One of the most important factors that affects the performance of computation and caching processes is the availability of bandwidth. This allows them to handle large amounts of content efficiently. In order to effectively manage their networks, both cloud and users need to have the necessary edge servers. These servers can help them with content update policy management and broadcast planning.

By simultaneously optimizing service caching, request scheduling, and resource-allocation policies, an online distributed method combining edge caching and computing management is studied in Ref. [85]. It cost-effectively minimizes the time–average service response latency of randomly arriving service requests, leveraging its online and distributed nature to investigate real-time queue sizes and link states. To address the dynamic and unpredictable challenges of virtual cars, the authors utilize Lyapunov optimization, matching theory, and multiplier consensus alternating directions to solve the problem in an online and distributed manner. Specifically, by using fresh data at each time point, the selection algorithm saves critical storage space and maintains generality, but at the cost of additional resource usage.

For collaborative intelligent optimization in the IoV, energy consumption is another issue that needs to be taken into account. Gu et al. [86] proposed the energy efficiency cost-reduction issue to carry out computational offloading in the vehicle edge computing network in order to balance delay and energy usage. In addition, a distributed iterative method is created to deal with multivariable and time-varying channel conditions. Transmission power, offloading and uploading times, decision-making, and processor frequencies are concurrently adjusted to establish a non-local efficient offloading method, which increases computation flexibility while considering the impact of vehicle motion on communication quality.

In order to strike a balance between energy consumption and delay, it is necessary to reasonably schedule the computation tasks requested by users in the vehicle–edge–cloud cooperative system. Ning et al. [89] studied the problem of task scheduling and energy consumption minimization among MECs when MEC devices are deployed on RSUs, and provided a processing model. Considering the power consumption of RSUs and EVs, task offloading based on the connection between the remaining power and the application is the method proposed by Zhai et al. [90]. Specifically, the cost function is established using the weighted sum of MEC energy consumption and the response time of the application, while combining SDN and fog computing to optimize the computational task. In the MEC–cloud collaboration, Michailidis et al. [91] made the Unmanned Aerial Vehicle (UAV) the air relay, combined with the ground RSUs deploying MEC to process the vehicle’s computing tasks, which reduced the processing delay. However, this may increase the energy consumption, so they proposed a multivariate optimization method to efficiently manage the energy of battery-supported RSUs and EVs. Energy issues and sustainable development are significant parts of IoV, which are crucial to the reliability and effectiveness of the system, and will continue to receive attention [92].

In the vehicle–edge–cloud collaboration, some researchers introduce digital twins (DT) to solve the problem of computational task offloading and resource allocation. DT technology establishes a real-time updated and interactive digital copy of the physical world, called a virtual model, which can be applied to mine information and simulate processes to obtain favorable results and feedback on the physical model, so as to achieve the purpose of optimization or provide new visualization services [93]. Liu et al. [94] adopted the power of digital twins to intelligently screen edge devices and offload tasks. Specifically, in the DT network, blockchain technology is utilized to ensure the consistency of information, and then computing devices with good communication quality and reliability are selected to allocate tasks. In order to solve the offloading balancing problem in edge computing of IoV, Xu et al. [95] chose edge computing devices as agents to apply deep reinforcement learning on the basis of DT technology, and the experiment verified that the proposed method could improve the level of service QoS. Another study [96] combined digital twins, game theory, and a distributed incentive mechanism to solve the problem of computing resource allocation from the perspective of energy efficiency and user recognition.

### 4.2. Data Storage and Cache Management in IoV

Like computing resources, the distribution of storage resources in the IoV is also uneven and increasingly unable to meet the needs of various large data services under collaborative intelligence. Both vehicles, RSUs, and MEC servers have limited storage space. Therefore, it is necessary to dynamically optimize storage data and cache management by fully considering the capacity and usage of storage resources of each node when computing tasks are uninstalled.

In Ref. [87], a cache service registry on mobile entities and a new metric to evaluate the effectiveness of service discovery are proposed. Caching the service registry makes the content discovery process less interactive with the RSUs. The cached contents of vehicles are available to neighbors. This kind of vehicle cache sharing can improve the discovery rate of accessible services and the utilization rate of services. When integrated into the RSUs content caching scheme, users can switch from edge caching to vehicle content acquisition based on density and mobility. This allows for more flexibility in design and is a great advantage for V2X environments.

Qiao et al. [88] proposed a collaborative edge caching scheme, which is a shared framework for communication, computation, and caching. It co-optimizes content placement and delivery through flexible trilateral cooperation among macro cell stations, roadside units, and intelligent vehicles in vehicle edge computing and networking. Data are cached and shared around infrastructure such as vehicles and RSUs in the form of multiple queues. Different strategies are designed for different time scales. The deterministic policy gradient deep learning algorithm is used to compute the optimal solution, which encourages and optimizes content sharing and reduces latency and packet loss.

In the IoV in the smart city scenario, Xu et al. [79] designed E-cache, an edge content caching framework including road side units and base stations. Combined with the deep spatiotemporal residual network, the traffic flow is predicted, and then the future service demand is predicted. This process can be divided into two stages: (1) the historical traffic flows are arranged into traffic flow vectors by a specific data embedding method and then spliced into tensors with different time dependencies. The future service demand is predicted after predicting the future traffic flow through the deep spatiotemporal residual network. (2) Initialize the edge content caching scheme according to the above selection, then modify the crossover and mutation, and select the reference point to get the optimal edge content caching scheme with the shortest executive time and lowest energy usage.

## 5. Collaborative Learning Technologies

Machine learning and AI have infiltrated various research and application fields since the Renaissance caused by backpropagation algorithms in the 1980s. AI technology with machine learning methods provides more intelligent solutions to improve the efficiency of IoV. With the development of the field of AI, many excellent machine learning methods have been derived, and also applied to the IoV for intelligent decision-making, which can be optimized for different related problems and needs. For example, unsupervised learning, supervised learning, deep reinforcement learning, and federated learning are used to solve specific problems such as path planning, communication routing protocol, task offloading, load balancing, vehicle blockchain, network attack prevention, and resource management based on traffic prediction.

Federated learning is a distributed machine learning method. Under the guidance of a central server, multiple clients use local data to train a model, then aggregate the model parameters of each client in the central server to integrate the knowledge, and then distribute it to each client. Since only intermediate model parameters are sent to the server instead of raw local user data, this is a way to obtain global knowledge under the premise of protecting user privacy.

Ensemble learning completes the learning task by constructing and combining multiple learners; that is, combining multiple weakly supervised models in order to obtain a better and more comprehensive strongly supervised model. The underlying idea of ensemble learning is that even if a weak classifier makes an incorrect prediction, other weak classifiers can correct the error back.

**Figure 6:**
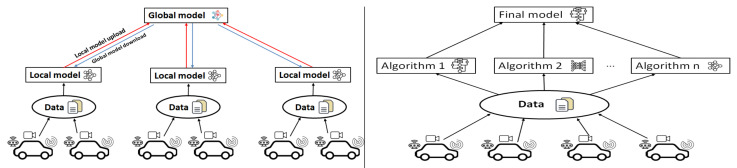
Learning parallelism (left: data parallelism, the same model; right: model parallelism, different models).

As shown in Figure 6, both federated learning and ensemble learning are distributed learning methods. They are all parallel learning models. However, the difference is that federated learning has the same model regardless of client or server, while ensemble learning has different models. Note that the ensemble learning uses multiple models to generate a better decision, where the data distributed to different models could be different or the same.

In the following, we provide a detailed discussion of the key learning approaches for collaborative intelligence in IoV, including:

- Federated learning.
- Ensemble learning.
- Supervised learning.
- Reinforcement learning.

### 5.1. Federated Learning for IoV

Lim et al. [97] discuss FL in detail from the perspectives of communication cost, resource allocation, security and privacy, and applications in mobile edge computing, and point out the challenges and feasible research directions. They believe that FL has strong optimization potential in mobile edge computing, and FL is an important scheme used to realize collaborative machine learning by transforming the centralized cloud computing model into a distributed edge–cloud collaborative computing.

Abreha et al. [98] study the routing protocol, framework, architecture, and hardware requirements of FL, then illustrate its feasibility through two application cases, and explain its advantages and challenges in edge computing from the perspective of engineering applications. The conclusion they come to is that FL is highly suitable for edge computing tasks because it utilizes the data and computing power of edges effectively.

Pokhrel and Choi [99] conduct a theoretical analysis about the latency and loss performance for multiple TCP connections in a Wi-Fi-available vehicular environment, and introduce a FL method to maximize system performances while maintaining the fairness among different TCP flows. Learning models are installed in vehicles, and the learning results are sent to a federal coordinator that is connected to vehicles through RSUs.

Xiong et al. [100] discuss the delay-aware task offloading problem in vehicular edge computing environments, where different types of communication approaches—namely dedicated Short Range Communication (DSRC), cellular vehicle-to-everything (V2X) communication, and millimeter wave (mmWave) communication—are available. In order to efficiently utilize the available communication and computing resources to minimize the task execution delay, they propose a FL-based approach to learn the best allocation policy in complex vehicular environments. Q-learning is used at each FL client to find the local best policies, and the learned results from multiple FL clients are aggregated to deal with different conditions. They use computer simulations to show that the FL-based approach can achieve a higher offloading success rate and better utilization of the communication and computing resources compared to existing baselines.

Fadlullah and Kato [101] consider a aerial–terrestrial networking scenario where the UAVs serve as base stations. They first introduce a distributed heterogeneous computing platform (HCP) integrating UAVs and terrestrial base stations. Then, they propose a two-stage federated learning algorithm to enhance the content caching process. The first stage of local training is conducted at mobile devices, and the second stage of training is conducted at UAVs. HCP aggregates the local models from mobile devices and UAVs to generate the global model.

Zhang et al. [102] discuss the use of federated learning to estimate the intelligence level of computer systems. They propose a vertical federated learning framework to conduct an intelligence ranking of autonomous vehicles without disclosing user data. Experimental results show that the FL-based approach outperforms existing baselines with acceptable time efficiency on both real and simulated data.

Yu et al. [103] discuss the joint communication, computing, and caching resource allocation problem for Ultradense edge computing environments, including smart vehicle systems. They design a DRL approach to minimize the task offloading delay and network resource consumption. A FL approach is also used to train the DRL model in a distributed manner for the purpose of data privacy protection. Experimental results demonstrate the effectiveness of both the DRL and FL.

Shiri et al. [104] consider the control problem of a massive population of UAVs in communication-resource-limited environments. A FL approach is used to distribute the learning process. One UAV is selected as the central unit; the other UAVs are used as FL clients. A neural network model is used at each FL client (UAV). The selection of the central unit is not discussed.

Sun et al. [105] integrates FL technology with digital twin technology for air–ground networks where a drone works as the central server of FL, and ground terminals work as FL clients. Digital twin technology is used to capture network dynamics in the training of the model. Numerical results are used to show the significance of the proposal in terms of learning accuracy and energy efficiency.

In Ref. [106], a FL approach is discussed for image processing applications in vehicular IoT. In addition, a model aggregation method is introduced to improve the accuracy of the global model and minimize the communication overhead for model exchanges. The model aggregation method takes into account the quality of local images, and the computational capability on each candidate for FL client.

Zhang et al. [107] propose a FL-based image classification system for multi-UAV networks. In this system, each UAV updates its local model based on the data from on-board cameras. A ground fusion center aggregates the local machine learning models generated by multiple UAVs to form a global model. The authors use computer simulations to demonstrate that the FL approach is able to reduce the communication cost significantly for a given target test accuracy, compared with the centralized training approach.

Saputra et al. [108] propose a FL-based energy demand prediction method for electric vehicle (EV) networks. In this method, charging stations are the FL clients, and the smart grid provider is the central server aggregating local models. A deep neural network is used as the machine learning model. In addition, taking advantage of the characteristic that charging stations in close geographical locations always have similar numbers of transactions, and similar transaction parameters, they also employ a clustering approach to generate CS clusters in order to reduce the impact of biased prediction results and improve the prediction accuracy.

In Ref. [109], Liu et al. introduce an aerial–ground integrated air-quality-sensing framework using FL based on UAV swarms. This framework includes aerial UAV swarms and ground-sensing systems, where the aerial-sensing part uses UAV cameras and the ground-sensing part uses static monitoring stations. In order to enable collaborative aerial sensing among UAVs from different agencies, UAVs work as FL clients to conduct local training, avoiding the transmission of camera data to other entities. A CNN model is used to learn the mapping between camera data and air-quality level. An UAV is used as the central server, which aggregates the learned model from multiple FL clients.

Thorgeirsson et al. [110] discuss the use of FL in energy demand and driving range prediction for electric vehicles. They consider two different types of learning models, namely, linear regression and neural network. EVs perform training of machine learning models based on local data, and the backend infrastructure (cloud) aggregates the local models to generate the global model. Different driving behaviors are considered by grouping drivers into multiple groups with a clustering technology, where each group defines its own model. They also discuss the effect of different extensions of the federated averaging algorithm on the FL performance.

Wang et al. [111] propose a secure federated learning framework for UAV-assisted crowdsensing applications. The UAVs train local models and send the models to a mobile edge computing (MEC) server, which could be installed on a base station for aggregation. Upon reception of a sensing task request, the MEC server selects a set of UAVs to perform the task. The framework can enable better utilities for UAVs than existing baselines.

Chai et al. [112] propose a hierarchical FL framework for knowledge sharing in IoV based on blockchain technology. Considering the diversity of driving routes and road environments, a hierarchical blockchain is introduced to improve the scalability of the proposed framework in terms of the network size. There are two types of FL clients and two types of blockchains, namely, vehicle client, RSU client, ground chain, and top chain, respectively. First, vehicles act as vehicle clients to train local models and then send the model updates to nearby RSUs. Then, RSUs collaborate with each other to record the model updates to the ground chain. RSU clients use the knowledge stored in the ground train and their own observations to train their models. RSU clients then record the learning results from both RSU clients and vehicle clients to the top chain, for the purpose of exchanging the information with other entities.

Lu et al. [113] propose a scheme for data sharing for IoV based on FL and blockchain technologies. A blockchain approach is employed to ensure the security. Based on the blockchain, an asynchronous FL is introduced to take into account the capability difference in FL clients. The central server selects the FL clients and the corresponding local training parameters in an adaptive approach by considering the capability of FL clients and the dynamic environments.

Yu et al. [114] propose a mobility-aware FL approach for content caching in IoV. Vehicles exchange content caching experience with each other using a FL-based approach, where vehicles are FL clients and RSUs are central servers. Each FL client trains a neural network based on local data related to content caching, and sends the learned experience to a RSU in proximity. A mobility-aware FL client selection approach is also proposed to ensure high-mobility training topology. The client selection considers the channel quality, connection time, and the importance of local data.

Lim et al. [115] propose a FL-based approach to enable distributed machine learning for UAV-Enabled IoV scenarios, including vehicle traffic prediction and parking slot management. A model owner allocates training tasks to UAVs. The whole geographical area is divided into multiple subregions, where each region is allocated to one UAV for training. A multi-dimensional contract-matching-based incentive mechanism is also designed to facilitate the participation of more candidates in the client selection.

Mowla et al. [116] propose a FL-based jamming defense mechanism for flying ad hoc networks. A Q-learning model is used in distributed learning of the best defense policy. Possible movements of UAVs are defined as the actions. UAVs learn the best defense policy by interacting with environments, and the local models are aggregated at an edge computing server such as a base station. By sharing Q-values among multiple UAVs with FL, the convergence speed at each vehicle can be reduced.

Pokhrel and Choi [117] design a blockchain-based decentralized FL approach for IoV. Vehicles act as FL clients, and exchange information with other network entities through a blockchain. The model updates from multiple FL clients are also aggregated by the blockchain network. Without discussing the details of the FL scheme, Ref. [117] discusses the effect of blockchain parameters and wireless channel parameters on the system-level performance.

There are also some studies discussing underlying technologies that better support FL in vehicular environments. Pham et al. [118] consider a FL scenario for mobile devices with limited battery supplies. They propose using UAVs to transfer power to mobile devices to enable sustainable FL-based wireless systems. They focus on UAV resource allocation and UAV placement. Rihan et al. [119] consider some FL-enabled use cases for vehicular IoT, and discuss fog computing architecture for V2X networks. Based on some discussion about the use of deep reinforcement learning (DRL)-based resource allocation and task offloading in vehicular fog computing environments, they also explore the possibility of using FL algorithms to improve the reliability of V2V communications. In Ref. [120], Yang et al. discuss the FL client scheduling problem in mobile networks involving smart phones or vehicles. They develop an analytical model to describe the performance of FL in wireless systems.

Yang et al. [121] propose an over-the-air computation approach to enabling FL in a wireless multiple-access channel. They propose a sparse and low-rank modeling method to facilitate the communication between the FL clients and the central server, allowing more clients to upload their model successfully.

Ng et al. [122] consider a UAV-enabled IoV scenarios where UAVs work as relay nodes to help the communication between a FL client (vehicle) and the central server. They propose a joint auction–coalition formation framework to incentivize UAVs in the forwarding of model updates.

### 5.2. Ensemble Learning for IoV

Jiang and Yin [123] propose a fault detection approach for vehicular cyber–physical systems based on an ensemble learning scheme. An accurate fault diagnosis requires the support of a large amount of data. However, these data are from different types of sensor devices and show different values in different weather and road conditions. Therefore, the fault detection should consider the variety of sensor data and environments. To conduct a more accurate diagnosis in various conditions, Jiang and Yin use an ensemble learning scheme to improve the adaptability of the fault diagnosis. The procedure of the scheme is as follows. First, different types of sensor information are collected through multiple sensors. Second, the data are processed by multiple models, where each model can recognize a special environmental condition. While each model reflects a limited feature of the whole system, by using the ensemble learning strategy to combine the results from multiple models, the scheme is able to enable more efficient data-driven fault diagnosis in complex vehicular environments.

Alamgir et al. [124] discuss the use of ensemble learning in finding the best modulation and coding scheme (MCS) for communications between underwater vehicles and underwater base stations. They employ the boosting technique, a type of ensemble learning, to analyze a measured sea trial dataset that includes channel parameters under different conditions. They further combine the regression tree technique with a boosting technique to find the best mapping between the MCS level and channel parameters. They claim that their approach can achieve 99.97% accuracy in classifying MCS levels.

Valle et al. [125] use random forests (RF), a well-used ensemble learning method, to better satisfy different service requirements, including extra-low latency and large throughput, in different vehicular scenarios. They consider a heterogeneous network scenario that includes multiples types of networks, including cellular communications through a base station, cellular V2V, and IEEE 802.11p. The objective of the ensemble learning method is to find the best available network according to the current vehicular environment and application requirements. The RF algorithm constructs multiple trees, where each tree is generated by parts of parameters (features) of the data. By using the RF algorithm, Ref. [125] is able to build an efficient system by making a decision based on the knowledge from multiple trees, which is not possible by a single simple algorithm.

Zhang et al. [126] use an ensemble learning approach in real-time driver behavior recognition tasks. They employ a deep CNN with multiple inputs, namely, side video streams, side optical flows, front video streams, and front optical flows, where each input is a weak classifier but has its own advantages in extracting some special features. By merging four different classifiers with the ensemble learning approach, Ref. [126] can improve the classification accuracy compared with existing baselines with low computational complexity.

Making a good prediction about the future network status and user traffic patterns is essential when it comes to providing successful network resource allocation in complex environments. In Ref. [127], Ferreira et al. discuss the use of ensemble learning in forecasting network performance metrics for fifth-generation (5G) networks. They propose a real-time distributed framework that uses multiple algorithms to make better forecasting. They show that an ensemble of multiple good algorithms can achieve a better prediction for different performance metrics. A vehicular network dataset in Porto, Portugal, is used to evaluate the performance of the framework.

Raja et al. [128] use an ensemble learning approach to enable an efficient intrusion detection system in vehicular ad hoc networks (VANETs). They consider the privacy protection of vehicle data and propose a collaborative approach based on private classifiers that are installed on each vehicle. While each vehicle is a weak classifier, using the communications among neighboring vehicles, the results from multiple vehicles are merged with an ensemble learning method, resulting in a much better intrusion detection system. Simulation results show that the ensemble classifier can achieve 96.94% accuracy without violating the user’s privacy.

In Ref. [129], an ensemble learning approach is applied in the path prediction of obstacle vehicles for the purpose of enabling a more advanced driver assistance system. Three different types of sensor data—namely, light detection and ranging (LiDAR), camera, global positioning system (GPS)—are considered trained by the ensemble learning. A recurrent neural network (RNN) is combined with ensemble learning to capture temporal behaviors of vehicles and environments. The authors show that the ensemble learning can improve the robustness of prediction with a network model of small size, which ensures its validity in dynamic road scenarios.

Toprak et al. [130] discuss the pedestrian detection problem in railway driver support systems where existing object detection technologies do not work well. They propose a three-stage system based on ensemble learning. In the first stage, various non-deep feature-classifier models are used to generate bounding boxes. Based on the results of the first stage, the second stage applies multiple deep CNN models to generate multiple individual adapted classification results. The final stage employs an ensemble learning method to combine the individual results to find a better detection result. The ensemble-learning-based system can significantly improve the detection accuracy as compared with well-used existing models.

Zhao et al. [131] propose an ensemble-learning-based approach for short-term vehicle traffic prediction. They use an ensemble learning model with the long short-term memory (LSTM) technique. The approach consists of two stages. In the first stage, different LSTM models repressing different effects of different time lags on the prediction are trained separately. In the second stage, ensemble learning is used to determine the weight coefficient of each LSTM model, and combine different models to make the final decision. They apply six different highway traffic datasets from Seattle, USA, to show that the ensemble-learning-based approach could perform better than six existing traffic flow prediction models.

Zhang et al. [132] propose WBELA, a weight-based ensemble learning algorithm, for intrusion detection in heterogeneous vehicular environments by identifying abnormal messages in the controller area network bus network. They use support vector machine (SVM), K-neighborhood (KNN), and decision tree (DT) methods as the basic classifiers. First, the CAN messages are processed by the basic classifiers for preclassification. Based on the preclassification results, an ensemble learning algorithm is used to optimize the weight of each classifier in order to reach the maximal accuracy and minimal errors. Simulation results are presented to show the advantage of the algorithm as compared with existing baselines in terms of precision and false positive rate.

For the purpose of enabling context-aware computing of smartphone applications, Alotaibi [133] discusses the problem of transportation mode detection of smartphone users, and proposes a method based on an ensemble learning algorithm. The method uses three different basic classifiers, specifically, random forests (RF), gradient boosting (GB), and decision trees (DT). Each classifiers votes independently, and the ensemble learning algorithm makes a better prediction by taking the majority vote of these three classifiers. The simulation results show that the method can achieve better results compared with existing human activity recognition approaches to classifying different transportation modes, namely, walking, standing, riding a train, driving a car, and riding a bus.

Wang et al. [134] propose an ensemble-learning-based method for traffic congestion detection in complex scenarios. The method first uses multiple deep neural networks to extract features from the data. In order to consider different levels of features, including low-level, middle-level, and high-level features, hierarchical feature extraction networks are employed. After that, based on the results from multiple networks, an ensemble learning approach is used to further improve the detection accuracy and generalization ability. Simulation results demonstrate that the deep learning ensemble can achieve a better performance compared with single estimator in complex scenarios.

### 5.3. Clustering with Unsupervised Learning

While k-means algorithm or its extensions are widely used, there are also some other clustering approaches. Here, we first discuss the use of k-means algorithms for IoV, and then show the applications of other clustering techniques.

Yousif et al. [135] discuss a license plate (LP) recognition problem where multiple types of LPs, namely Arabic–Egyptian license plates and English license plates, are considered. A k-means clustering algorithm is applied to segment the LP characters. Experimental results show that their approach can achieve higher recognition accuracy than existing alternatives.

Pustokhina et al. [136] present a deep-learning-based license plate recognition model that uses a k-means algorithm in character segmentation, and uses CNN in character recognition. Experimental results on three different datasets show the advantage of the model compared with existing baselines.

Yang et al. [137] study the challenges in recognizing the decision-making style of a driver. They introduce an approach that integrates an unsupervised learning approach, specifically, a k-means algorithm, with a supervised learning approach, a k-nearest neighbor (KNN) algorithm. They demonstrate that their approach can improve the traditional KNN algorithm with 72.67% faster recognition time and can shorten the recognition time by more than 72.67%, with higher accuracy.

Rong et al. [138] discuss the challenges of simultaneous localization and mapping in mobile environments, and employ a k-means algorithm to conduct image segmentation for the purpose of better processing dynamic regions. They use experiments to demonstrate the validity of the approach in terms of trajectory accuracy and computing speed.

Bhaumik et al. [139] discuss the clustering problem of a channel impulse response for 60 GHz mmWave multipath channels in vehicular environments. Considering that the performance of the k-means algorithm is dependent on the value of k, they combine the k-means algorithm with a time–amplitude clustering algorithm to group multipath components based on their time delays and amplitudes.

Gedschold et al. [140] discuss a similar multipath clustering problem in vehicle-to-infrastructure (V2I) channels. They employ a variant of the k-means algorithm, namely, K-Power-Means, to conduct the clustering of multipath components. An algorithm is also used to find the best number for clusters before conducting the clustering.

Song et al. [141] study the multi-hop broadcast issue in VNs, which is considered to be useful for disseminating safety-related messages. They introduce a vehicle-clustering-approach-based k-means algorithm. The selected cluster heads are used as forwarder nodes in multi-hop broadcasting. The geographical position of vehicles and inter-vehicle distance are considered in the clustering algorithm. Since the signal quality and vehicle mobility are not discussed in the clustering algorithm, this clustering algorithm requires an enhancement for handling complex vehicular environments.

Sliwa et al. [142] discuss the data transfer problem in for delay-tolerant vehicular applications, such as environmental map construction, traffic monitoring, weather sensing, and so forth. They define some geographical regions as black spots where the transmission rate should be reduced due to insufficient communication capability. Based on vehicular big data, a k-means algorithm is used to conduct clustering of black spots.

Shehzad et al. [143] consider the use of UAVs to improve the communication performance between small cell base stations. The focus on the optimal placement of UAVs for the purpose of serving more small cell base stations. A k-means algorithm is used to solve the problem by considering multiple metrics such as the achievable data rate, available bandwidth, and the capability of UAVs. Numerical results are used to show the superiority of the algorithm over the conventional approach in terms of the achievable data rate and spectral efficiency.

Hoang et al. [144] consider a wireless authentication system that uses UAVs as relay nodes. They discuss the problem of how to detect eavesdropping attacks targeting UAVs. They build a detection model combing one-class support vector machines (OC-SVM) with k-means clustering. Numerical results are presented to show the proposed model in terms of stability.

Targeting for mobile communication systems for high-speed trains and vehicles, Albakay et al. [145] propose an improved k-means algorithm for estimate frequency offset from the in-phase and quadrature constellation of the received signal. They divide the received signal symbols into multiple groups and find the centroid of each group using the modified k-means algorithm. The algorithm is able detect the constellation rotation within a range between −45 degrees and 45 degrees for QPSK signals with additive white Gaussian noise.

He et al. [146] consider a drone-aided emergency communication network in post-disaster scenarios, and employ a modified k-means algorithm to solve the drone deployment problem with the aim of minimizing power consumption of drones while satisfying user requirements regarding the transmission speed. The k-means algorithm is used to find the optimal number, altitude, and transmit power of drones.

Ye et al. [147] discuss the effect of vehicle density on the communications in VANETs, and propose using a clustering approach to reduce the packet collisions in VANETs. They use an improved k-means algorithm to group vehicles into different clusters by considering the similarities in movements, vehicle location, and inter-vehicle distance. Based on the vehicle clusters, a message distribution mechanism is designed. Computer simulations are used to show that the mechanism can achieve a higher packet delivery ratio and lower latency compared with existing baselines.

Different vehicular applications possibly exhibit different levels of service requirements. Network slicing is a promising technology for fulfilling the service requirements involving a large amount of diversity. Cui et al. [148] propose a k-means++ algorithm to group the services into different clusters based on the service-level agreement—including the capacity, coverage area, and QoS requirements—between the service provider and customer. Based on the clustering results, different network slices are provided to different groups of services.

Ozasa et al. [149] consider a communication network where UAVs act as base stations, providing wireless communications services to other terminals. They discuss the joint UAV placement and frequency division problem in multi-UAV environments. A k-means algorithm is used to derive the best horizontal arrangement of UAVs. The frequency division is conducted after the clusters are generated.

Wahlstrom et al. [150] study the smartphone placement problem for smartphone-based driver monitoring. They use a kernel-based k-means clustering approach to infer the placement of smartphones within vehicles (the position of the smartphone with respect to the vehicle). They argue that knowing the placement of the smartphone could provide benefits in many scenarios, such as accident reconstructions and improving driving behaviors.

Yuan et al. [151] study the modeling of the driving cycle of a city tour bus. The driving cycle construction is useful for evaluating vehicle performance and measuring fuel consumption. As the conventional k-means algorithm is sensitive to its initialization parameters, an improved version of k-means—namely, GA-K-means—integrates a genetic algorithm. The GA-K-means algorithm groups micro-trips, specifically, an excursion between two successive time points at which the vehicle is stopped. They further combine GA-K-means with a hidden Markov model to construct the driving cycle of city tour buses in Beijing.

Forster et al. [152] study how to identify multiple characteristic driving cycles from vehicle data representing real driving scenarios. They use a k-means algorithm to find k groups of micro trips to represent different driving cycle features. They show that by choosing only one parameter, k, the k-means algorithm can achieve a scalable solution for different granularities of driving scenarios.

In Ref. [153], the density-based spatial clustering of applications with noise algorithm is employed to design an adaptive clustering scheme for low-power base stations in coordinated multipoint transmission and reception operation. They show that DBSCAN has advantages in identifying geographically isolated low-power base stations and improving system throughput compared with the k-means algorithm.

For the purpose of enabling more intelligent traffic monitoring, Cao et al. [154] discuss the lane determination of vehicles based on millimeter-wave radar data. They propose a kernel line segment adaptive possibilistic c-means clustering algorithm to achieve better clustering. KLSAPCM is able to conduct efficient clustering without knowing the position and angle of the radar. The experimental results are used to show the advantage of KLSAPCM in a lane determination problem over other clustering algorithms, including DBSCAN and k-means algorithms.

In Ref. [155], hierarchical clustering is employed for anomaly detection in VANETs. The anomaly detection is conducted at a roadside unit without revealing the content of user traffic. The anomaly detection also uses dynamic time warping in distance measurement.

Chen et al. [156] discuss the driving cycle prediction problem for hybrid electric vehicles. They employ a k-shape clustering algorithm to group the driving cycle data into six different groups. Simulation results are used to show that the k-shape algorithm performs better than k-means in this problem.

### 5.4. Reinforcement Learning

In order to achieve a more accurate understanding of the complex vehicular environments, a vehicle has to utilize the information received from other vehicles in the vicinity. However, the vehicle has to ensure that whether the messages received from other vehicles are trustworthy or not. If the messages include fake or misleading information, the perception accuracy degrades drastically, possibly resulting in a fatal error. Therefore, the trust management in vehicular environments is an important consideration for achieving a good decision. Guo et al. [157] propose a context-aware trust management model in VANETs. The context includes internal information, specifically the information collected from the local sensor (the sensor devices installed on the current vehicle), and external information received from other vehicles. A Q-learning-based approach is proposed to set the weights of the internal and external information in making the final decision. The information entropy theory is used in the trust calculation. Simulation results show that the model can achieve a higher precision for trust evaluation with reasonable commotional and communication overhead.

Multi-agent reinforcement learning (MARL) is widely used in vehicular IoT to enable an efficient system in a distributed manner. However, the coordination among vehicles in a dynamic environment is particularly challenging. Yu et al. [158] formulate the coordination problem by using a dynamic coordination graph. They propose coordination-graph-based MARL approaches to optimize the joint maneuvers of multiple vehicles. Experimental results are presented to show the performance of the proposed approaches in the decision making surrounding following or overtaking in highway situations to enable high-level strategic control of vehicles.

Zhou et al. [159] consider the decentralized control of multiple robots, such as UAVs, for patrolling in partially observable environments with unknown prior knowledge. For example, for a post-disaster scenario, while an UAV can patrol continuously over the disaster area and collect information by coordinating with other UAVs, the decentralized control of multiple UAVs is challenging due to the environment, which is unknown and only partially observable for each UAV. The authors formulate the problem as a Bayes-adaptive transition-decoupled partially observable Markov decision process, and propose a Bayesian RL algorithm to improve the coordination and planning process of multiple robots. The experimental outcomes indicate that the proposed algorithm performs better than the existing methods.

It is important to consider stability and efficiency in the design of a RL algorithm, especially for IoV applications, which require ultra-high reliability. Zhang et al. [160] propose a deterministic promotion RL algorithm approach that improves policy evaluation in critique and exploration in action. The action exploration in the proposed algorithm is conducted by a normalization-based evaluation approach in a way that can increase efficiency and decrease the dependencies among the explored actions. They use both computer simulations and real-world experiments to show the advantage of the proposed algorithm in a longitudinal velocity control scenario for vehicular environments.

In Ref. [161], Feng and Haykin discuss the jamming problem in vehicular networks. They investigate anti-jamming V2V communications based on joint control of power and channel selection. The problem is formulated as a multi-armed bandit problem, and a RL-based approach is tailored to solve the problem. Extensive simulations are conducted to show the performance of the approach in terms of multiple performance metrics, including power strategy, channel selection, and throughput.

Xing et al. [162] discuss the importance of trust evaluation in intrusion detection systems. They argue that the trust evaluation of vehicles should be conducted not only by RSUs, but also by the vehicles in the vicinity. However, in order to achieve a trust evaluation based on the collaboration of a large portion of vehicles, an efficient incentive mechanism is required. Therefore, the authors propose a RL-based incentive mechanism to involve the participation of more vehicles. They use computer simulations to show that the mechanism can achieve a higher detection ratio than the conventional approach.

Kapoor et al. [163] discuss the user association problem for small cell dense VNs, where a vehicle could possibly connect to multiple small cell base stations. The mobility of vehicles result in handovers between different base stations, which makes the user association policy—specifically, which base station to connect with at a particular time—very important. In Ref. [163], the authors propose a signal-quality-aware user association algorithm based on Q-learning, a form of RL. The algorithm is able to make a tradeoff between the number of handovers and system performance while guaranteeing the QoS requirements.

Zhou et al. [164] discuss the radio resource allocation at base stations in VNs. They define a Q-learning model where the state is the uplink and downlink data rate against aviate channel resource. The action is the ratio between the resource allocated for uplink and downlink data transmission. The reward is defined based on multiple network status parameters. By using the Q-learning algorithm, Ref. [164] is able to consider the future reward for a resource allocation policy, which can provide a solution with long-term efficiency.

Wu et al. [165] propose a RSU-assisted routing protocol for VANETs based on Q-learning. They use a three-phase routing algorithm. In the first phase, a packet is forwarded from the source node to a nearby RSU. In the second phase, the packet is forwarded between RSUs. In the last phase, the packet is delivered to the destination from a RSU to the destination. Hello messages are used to exchange information among neighbors and update Q-table.

Samir et al. [166] consider a scenario in which UAVs act as relay nodes to improve the coverage of cellular infrastructure. A reinforcement-learning-based approach is proposed to optimize the trajectories of multiple UAVs while minimizing the number of deployed UAVs and energy consumption. The learning is conducted at the central unit that interacts with other network entities to update the model. Numerical results are used to demonstrate the advantage of the RL-based approach over existing baselines.

Raja et al. [167] discuss the communication gateway selection problem in a multi-access vehicular environment where multiple types of radio access technologies, including Wi-Fi and cellular communication, coexist. They propose a Q-learning-based approach to support each vehicle to select the best gateway nodes among a cellular base station and multiple RSUs where RSUs are connected with vehicles through a Wi-Fi interface. In the learning model, the state is defined by the distribution of vehicles, and the action is the selection of the gateway. While each vehicle is a learning agent, the real learning process is conducted a centralized server, and the software defined network (SDN) technology is used to achieve centralized control of the entire vehicular network.

There are some studies which discuss the use of reinforcement learning in improving vehicle path planning. Cao et al. [168] propose a Q-learning algorithm to minimize the delay in transportation. In the Q-learning algorithm, the action is the driving direction, and Q-value represents the probability of satisfying the deadline constraint in transportation. They use both artificial transportation networks and real road networks in Beijing, Munich, and Singapore to evaluate the efficiency of the proposed algorithm.

Zhang et al. [169] propose a RL-based scheme for route planning for hybrid electric vehicles for the purpose of finding the route with minimum energy consumption. The RL model is defined as follows. The state is represented by a vector consisting of vehicle position, power demand, vehicle velocity, and the state of charge for battery. The action is represented by a vector that consists of output torque from engine, gear selection, and direction. The reward is defined based on the energy recuperation level. They conduct computer simulations by using Toyota Prius as the vehicle model, and evaluate the performance of the proposed approach for various navigation tasks.

## 6. Discussion of Future Research Directions

**Figure 7:**
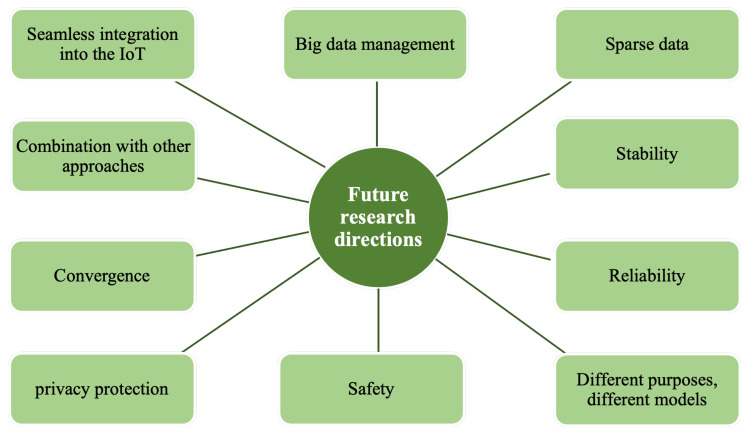
Several valuable future research directions of Iov with CI.

The rapidly growing paradigm trend of Internet of Vehicles predicts that it will gain attention and development in many fields and industries in the next decade. The main goal of applying collaborative intelligence ideas and technologies to the field of IoV is to integrate the data and resources of a large number of vehicles, users, infrastructure, and networks, providing systems with reliability and connectivity that are easy to manage, control, and operate. Due to the dynamic nature of vehicles, the diversity of devices, the low time delay requirements of applications, and the connectivity of V2X, various special requirements are put forward for the collaboration of this complex system. However, these aspects bring new technical challenges to the design and expansion of the IoV field. Important future research directions that will help to conquer those challenges are presented in Figure 7, as follows:

- Big Data Management: In IoV under collaborative intelligence, multiple types of smart devices generate a large amount of data, which are stored locally or in the cloud. Network latency and insufficient storage affect computation and analysis, and may even break the system. Therefore, the real-time management and analysis of IoV big data has always been a challenge, but will also be a challenge in the future. (1) Comprehensive awareness of the environment: Fusing sensor data and historical knowledge in the collaborative network provides comprehensive awareness of the environment for the whole system and each entity, so as to improve the accuracy and security of decision making. (2) Storage and preprocessing of big data: Effectively utilizing the storage capacity of edge nodes and cloud data centers to further compress the increasing massive data and eliminate a large amount of redundancy is another important research direction. (3) Data transmission and unified standards: In the case of vehicle movement and low latency, big data have more stringent requirements on communication, so reducing the amount of transmitted data and improving communication efficiency is inevitable. Research on more reasonable communication protocols, design of edge computing models with a small amount of data transmission, and use of semantic communication to compress data are all directions which researchers can make further efforts to explore. Furthermore, establishing a unified transmission standard and increasing investment in infrastructure can greatly accelerate the development of IoV.
- Sparse Data: Since most collaborative intelligence requires machine learning methods that are data-driven or interact with the environment, a large amount of training data will be used to train a model for a certain task. This puts forward high requirements for data collection and processing, but some tasks make it difficult to collect corresponding high-quality data in reality, resulting in the problem of data sparsity. In order to overcome this problem, many studies use realistic simulators to generate simulated data for model training, and then transfer to real data to fine-tune the model. On the other hand, the learning model is built and adjusted, or other intelligent methods are combined to optimize the training effect. In addition, expanding the scope of collaboration and deepening the cooperation in collaboration can alleviate the problem of data sparsity to a certain extent. This is also an aspect to which future researchers can devote their energy.
- Stability: Vehicles are dynamic in nature, and the network topology of IoV also changes at any time, so stability is also a major challenge in this field. The stability is reflected in the network connectivity, and the data and signal transmission between agents need high-quality communication capabilities. Although there are many research studies on the cooperative communication of Internet of Vehicles, the Internet facilities for Internet of Vehicles or Internet of Things are not perfect in most areas, and it will take some time to achieve coverage of 5G and the development of 6G. Therefore, the real realization of collaborative intelligent communication in the Internet of Vehicles still needs efforts.
- Reliability: Applications related to intelligent transportation and UAV detection are usually sensitive to safety, because such applications require high reliability, otherwise there will be serious losses of life and property. In the application of Internet of Vehicles, reliability is an important issue due to the large scale of the network, complex computing architecture, and poor network stability.
- Different Purposes, Different models: Collaborative intelligent models based on machine learning are widely used in Internet of Vehicles tasks and have achieved good results, such as reinforcement learning, federated learning, centralized learning, etc. However, in different tasks and objectives, different learning models and methods are usually used. The data demand is large, the processing is tedious, the model parameters are particularly large, the training time is long, and the model is not universal. In future research, this problem can be optimized from two perspectives. (1) Building a unified large model that integrates data and is suitable for multi-class tasks after a simple fine-tuning process. (2) Constructing different models for different purposes to refine tasks and achieve better results.
- Safety: Safety is one of the core challenges in the field of Internet of Vehicles. It is a network that is connected by a variety of devices and integrates different technologies and standards through the Internet to achieve collaborative intelligence. Not only must infrastructure such as roadside units, cloud storage centers, and computing centers be connected to the network, but also most of the vehicle’s devices such as GPS, cameras, sensors, brakes, and accelerators may be accessed remotely. So if the network security is not in place, the attacker may control a large number of network devices or even directly control the vehicle, leading to serious consequences. At present, IoV is not completely secure against all kinds of attacks, such as quantum attacks. Therefore, the discovery and repair of security vulnerabilities, and even the establishment of a more perfect security system are indispensable challenges in the future.
- Privacy Protection: In V2X network, the everything-connected network will create the problem of data and privacy exposure. How to protect user privacy and data is also a very recent concern, including the application of federated learning and blockchain technology in IoV collaboration. The most popular solution in this regard is to use federated learning and blockchain technology, which can avoid the direct transmission of original user data to make task decisions and take advantage of the decentralization and tamper-proof characteristics of blockchain to improve the privacy protection ability. However, the cost of applying these technologies in IoV collaboration is not low, and there are unsolved difficulties.
- Convergence: The problem of training efficiency of machine learning models is often involved in coordinated intelligence methods. Under the strict service requirements of high security, stability and low latency, methods that help the model achieve higher learning accuracy and faster convergence speed are worthy of further improvement.
- Combination with other approaches: At present, the method of deep learning is widely used to realize the collaborative intelligence of Internet of Vehicles, and has achieved good results. On this basis, other effective ideas and approaches, such as fuzzy logic, semantic communication, and digital twins, can be combined to further optimize the task results.
- Seamless integration into the IoT in future: As a part of the future Internet of Things, the Internet of Vehicles must be integrated into the smart city and other next-generation Internet of Everything environment, such as Industry 5.0. Therefore, we believe that the development of IoV technology and the deployment of practical applications should also consider integration and collaboration with other fields, such as smart healthcare. At the same time, the collaboration with human needs to be paid more attention to lay the foundation for providing users with more personalized services.

## 7. Conclusions

With the development of Artificial Intelligence, distributed computing, supercomputers, hardware such as GPU and other technologies, autonomous driving under the Internet of Vehicles, and even smart cities under the Internet of everything will certainly become a reality in the near future. In the following emerging applications and services, collaborative intelligence will play an important role because it can integrate the knowledge and capabilities of multiple smart devices, including data, communication, computing, and storage resources. With the increasing interest in collaborative intelligence schemes from both industry and academia, it is particularly important to explore their research in the context of vehicle-borne IoT. In this paper, we discussed the existing research, technical challenges, possible solutions, and open problems of CI for IoV applications. This paper first briefly introduced IoV and the CI problem in IoV, and then expounded its advantages and challenges in the wireless Internet of Things environment. Then, from the aspects of collaborative communication, collaborative computing, and collaborative learning methods, the existing research on the application of CI methods in vehicle-borne Internet of Things was reviewed, and the technical problems were discussed. Finally, the future research direction of the integration of CI and IoV was discussed to accelerate the research process of IoV.

## Data Availability

Not applicable.

## Abbreviations

The following abbreviations are used in this manuscript:

AC	Actor–Critic.
AI	Artificial Intelligence.
CI	Collaborative Intelligence.
CNN	Convolutional Neural Network.
C-V2X	Cellular Vehicle to Everything.
DSRC	Dedicated Short Range Communication.
DT	Decision Tree.
EV	Electric Vehicle.
GB	Gradient Boosting.
GPS	Global Positioning System.
HCP	Heterogeneous Computing Platform.
HVN	Heterogeneous Vehicle Network.
IoT	Internet of Things.
IoV	Internet of Vehicles.
ITS	Intelligent Transportation System.
IV	Intelligent Vehicles.
KNN	K-Neighborhood.
LiDAR	Light Detection and Ranging.
LP	License Plate.
LSTM	Long Short-Term Memory.
MARL	Multi-Agent Reinforcement Learning.
MCS	Modulation and Coding Scheme.
MEC	Mobile Edge Computing.
MIMO	Multiple-Input Multiple-Output.
mmWave	Millimeter Wave.
NFC	Near Field Communication.
NSs	Network Slices.
QoS	Quality of Service.
RAT	Radio Access Technology.
RF	Random Forests.
RSU	Roadside Unit.
SDN	Software Defined Network.
SVM	Support Vector Machine.
UAVs	Unmanned Aerial Vehicles.
V2I	Vehicle-to-Infrastructure.
V2N	Vehicle-to-Cloud/Edge.
V2P	Vehicle-to-Pedestrian And Cyclist.
V2R	Vehicle-to-Road.
V2V	Vehicle-to-Vehicle.
VANET	Vehicular Ad Hoc Network.
VN	Vehicular Network.
WLAN	Wireless Local Area Network.
